# Supplementary material for: Comparison of Different Dietary Fatty Acids Supplement on the Immune Response of Hybrid Grouper (Epinephelus fuscoguttatus × Epinephelus lanceolatus) Challenged with Vibrio vulnificus
Source: Biology (Basel). 2022 Aug 30;11(9):1288. doi: 10.3390/biology11091288 (PMC9495948; doi:10.3390/biology11091288)
Supplement: Supplementary file 1 [file biology-11-01288-s001.zip › Supplementary Figure S1.pdf]

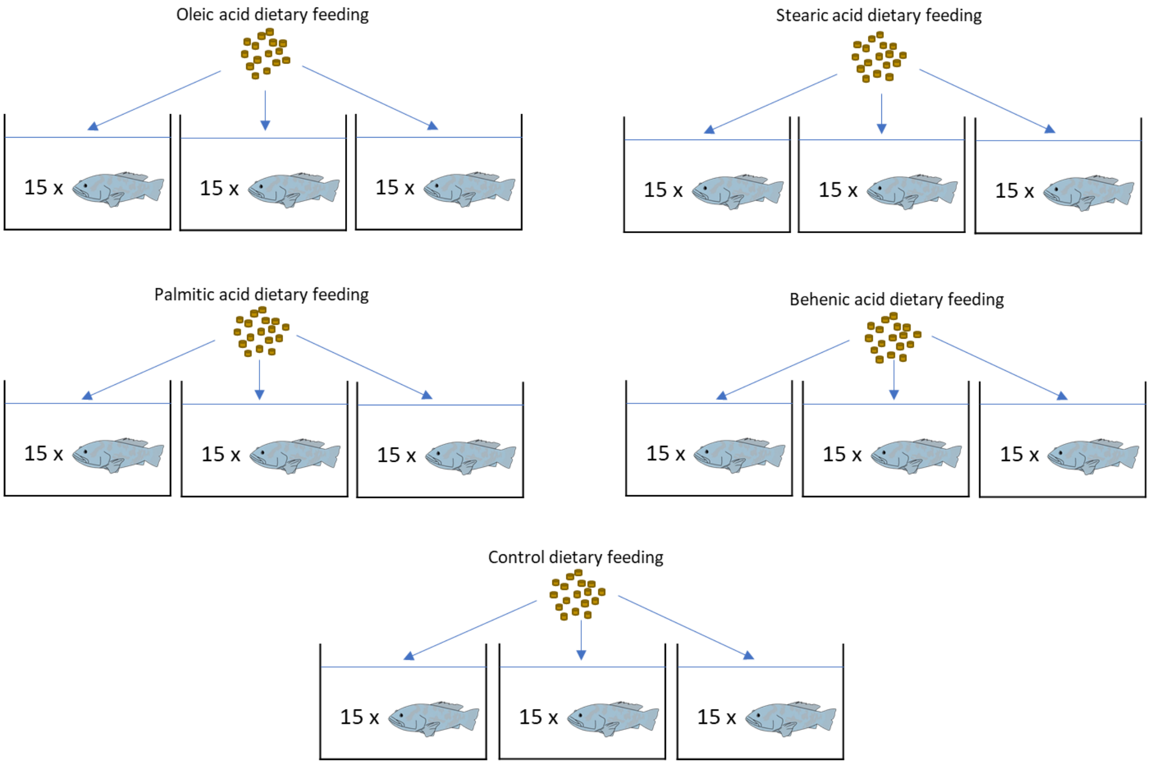

Supplementary Figure S1. Diagram of glass aquariums set up for five different fatty acid feeding experiments in three replicates.
