# Supplementary material for: Comparison of Different Dietary Fatty Acids Supplement on the Immune Response of Hybrid Grouper (Epinephelus fuscoguttatus × Epinephelus lanceolatus) Challenged with Vibrio vulnificus
Source: Biology (Basel). 2022 Aug 30;11(9):1288. doi: 10.3390/biology11091288 (PMC9495948; doi:10.3390/biology11091288)
Supplement: Supplementary file 1 [file biology-11-01288-s001.zip › Supplementary Figure S2.pdf]

>*Vibrio vulnificus* isolate 9 16S ribosomal RNA gene, partial sequence
Sequence ID: AY245189.1 Length: 1250
Range 1: 385 to 1083, Score:1242 bits (672), Expect:0.0,
Identities:691/700(99%), Gaps:2/700(0%), Strand: Plus/Plus

```
Query 2     AGCACCGGCTAACTCCGTGCCAGCAGCCGCGGTAATACGGAGGGTGCGAGCGTTAATC-G   60
            |||||||||||||||||||||||||||||||||||||||||||||||||||||||||| |
Sbjct 385   AGCACCGGCTAACTCCGTGCCAGCAGCCGCGGTAATACGGAGGGTGCGAGCGTTAATCGG   444

Query 61    GAATTACTGGGCGTAAAGCGCATGCAGGTGGTTTGTTAAGTCAGATGTGAAAGCCCGGGG   120
            ||||||||||||||||||||||||||||||||||||||||||||||||||||||||||||
Sbjct 445   GAATTACTGGGCGTAAAGCGCATGCAGGTGGTTTGTTAAGTCAGATGTGAAAGCCCGGGG   504

Query 121   CTCAACCTCGGAATTGCATTTGAAACTGGCAGACTAGAGTACTGTAGAGGGGGGTAGAAT   180
            |||||||||||||| |||||||||||||||||||||||||||||||||||||||||||||
Sbjct 505   CTCAACCTCGGAATAGCATTTGAAACTGGCAGACTAGAGTACTGTAGAGGGGGGTAGAAT   564

Query 181   TTCAGGTGTAGCGGTGAAATGCGTAGAGATCTGAAGGAATACCGGTGGCGAAGGCGGCCC   240
            ||||||||||||||||||||||||||||||||||||||||||||||||||||||||||||
Sbjct 565   TTCAGGTGTAGCGGTGAAATGCGTAGAGATCTGAAGGAATACCGGTGGCGAAGGCGGCCC   624

Query 241   CCTGGACAGATACTGACACTCAGATGCGAAAGCGTGGGGAGCAAACAGGATTAGATACCC   300
            ||||||||||||||||||||||||||||||||||||||||||||||||||||||||||||
Sbjct 625   CCTGGACAGATACTGACACTCAGATGCGAAAGCGTGGGGAGCAAACAGGATTAGATACCC   684

Query 301   TGGTAGTCCACGCCGTAAACGATGTCTACTTGGAGGTTGTGGCCTTGAGCCGTGGCTTTC   360
            ||||||||||||||||||||||||||||||||||||||||||||||||||||||||||||
Sbjct 685   TGGTAGTCCACGCCGTAAACGATGTCTACTTGGAGGTTGTGGCCTTGAGCCGTGGCTTTC   744

Query 361   GGAGCTAACGCGTTAAGTAGACCGCCTGGGGAGTACGGTCGCAAGATTAAAACTCAAATG   420
            ||||||||||||||||||||||||||||||||||||||||||||||||||||||||||||
Sbjct 745   GGAGCTAACGCGTTAAGTAGACCGCCTGGGGAGTACGGTCGCAAGATTAAAACTCAAATG   804

Query 421   AATTGACGGGGGCCCGCACAAGCGGTGGAGCATGTGGTTTAATTCGATGCAACGCGAAGA   480
            ||||||||||||||||||||||||||||||||||||||||||||||||||||||||||||
Sbjct 805   AATTGACGGGGGCCCGCACAAGCGGTGGAGCATGTGGTTTAATTCGATGCAACGCGAAGA   864

Query 481   ACCTTACCTACTCTTGACATCCAGAGAACTTTCCAGAGATGGATTGGTGCCTTCGGGAAC   540
            ||||||||||||||||||||||||||||||||||||||||||||||||||||||||||||
Sbjct 865   ACCTTACCTACTCTTGACATCCAGAGAACTTTCCAGAGATGGATTGGTGCCTTCGGGAAC   924

Query 541   TCTGAGACAGGTGCTGCATGGCTGTCGTCAGCTCGTGTTGTGAAATGTTGGGTTAAGTCC   600
            ||||||||||||||||||||||||||||||||||||||||||||||||||||||||||||
Sbjct 925   TCTGAGACAGGTGCTGCATGGCTGTCGTCAGCTCGTGTTGTGAAATGTTGGGTTAAGTCC   984

Query 601   CGCAACGAGCGCAACCCTTATCCTTGTTTGCCAGCGAGTAATGTCGGGAACTCCAGGGAG   660
            |||||||||||||||||||||||||||||||||||   |   ||  ||||||||||||||||
Sbjct 985   CGCAACGAGCGCAACCCTTATCCTTGTTTGCCAGCACTTCGGGT-GGGAACTCCAGGGAG   1043

Query 661   ACTGCCGGTGATAAACCGGAGGAAGGTGGGGACGACGTCA   700
            ||||||||||||||||||||||||||||||||||||||||
Sbjct 1044  ACTGCCGGTGATAAACCGGAGGAAGGTGGGGACGACGTCA   1083
```

Figure Supplementary S1. The 16S rDNA of partial nucleotide sequence comparison using BLAST in NCBI Genebank database
